# Hemodynamic effects of hemodialysis: the interaction between the heart and the arteries

**DOI:** 10.1007/s40620-025-02434-y

**Published:** 2025-10-07

**Authors:** Aidana Mustafa, Aigerim Yermekbay, Aizhan Zhankorazova, Bauyrzhan Toktarbay, Zaukiya Khamitova, Dinara Jumadilova, Alessandro Salustri

**Affiliations:** Department of Medicine, Nazarbayev University School of Medicine, 5/1 Kerey and Zhanibek Khans Str., 010000 Astana, Kazakhstan

**Keywords:** Hemodialysis, Left ventricular function, Arterial stiffness, Ventriculo-arterial coupling

## Abstract

Many studies report that cardiac function is affected by hemodialysis due to alterations in left ventricular morphology and function, particularly left ventricular hypertrophy. Left ventricular hypertrophy is primarily driven by pressure and volume overload, aggravated by factors such as arteriovenous fistulas, anemia, and fluid retention. In addition to left ventricular mass, hemodialysis can impair both left ventricular systolic and diastolic functions, leading to transient reductions in left ventricular ejection fraction, and global longitudinal strain, which are strongly linked to increased mortality. Moreover, chronic dialysis leads to changes in arterial structure and function, including increased intima-media thickness and reduced arterial distensibility, which result in increased afterload. Fluctuating blood pressure during dialysis further affects cardiac function, emphasizing the need for comprehensive assessment of both ventricular and arterial functions, a relationship defined as ventriculo-arterial coupling. In patients with kidney failure, ventriculo-arterial coupling serves as a valuable load-independent prognostic marker, enhancing risk prediction and stratification. Non-invasive tools like echocardiography and speckle-tracking techniques are currently available for evaluating these parameters, enabling early detection and intervention to mitigate cardiovascular risks in patients with kidney failure undergoing hemodialysis. These insights highlight the complex interplay between fluid management, left ventricular function, and arterial stiffness, emphasizing the importance of improved strategies to optimize cardiovascular outcomes in this high-risk population.

**Graphical abstract:**

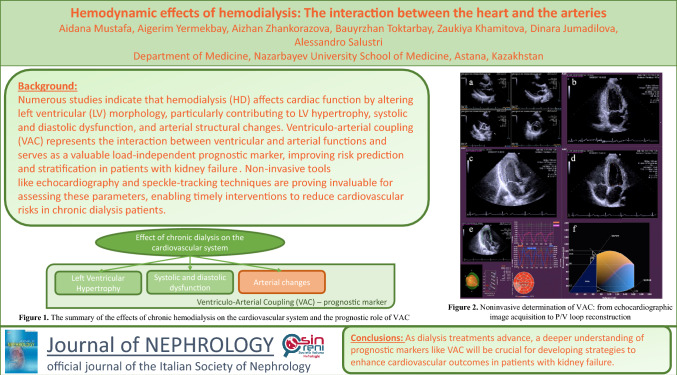

## Introduction

Patients undergoing hemodialysis (HD) often face significant cardiovascular challenges, which can dramatically affect their long-term health outcomes [1, 2]. Among these challenges, alterations in left ventricular (LV) morphology and function are especially common, manifesting in various forms of cardiac remodeling such as LV hypertrophy (LVH). LVH is a critical marker of cardiovascular risk and survival in patients with kidney failure, with studies showing that nearly 80% of dialysis patients experience some degree of increased LV mass [3]. This hypertrophy is driven by a combination of pressure and volume overload, commonly exacerbated by factors such as arteriovenous fistulas, anemia, and fluid retention [4].

Beyond LV mass, HD can also impair both LV systolic and diastolic function. Several studies indicate that HD leads to a transient reduction in LV ejection fraction (LVEF), global longitudinal strain, and other measures of systolic function, highlighting the acute impact of HD on cardiac performance [5]. Furthermore, diastolic dysfunction, marked by impaired relaxation and filling of the LV, significantly worsens post-HD and is associated with increased mortality in dialysis patients [6]. A key contributor to diastolic dysfunction in these patients is myocardial ischemia, which occurs due to changes in blood pressure and impaired coronary perfusion during dialysis [7].

Arterial stiffness and the intimate relationship between the heart and the large arterial vessels are also significantly affected by chronic dialysis. Changes in arterial structure and function, including increased intima-media thickness and reduced arterial distensibility, contribute to elevated afterload and higher cardiovascular risk [8–11]. These changes in arterial stiffness are coupled with fluctuating blood pressure during dialysis, which further complicates cardiac function. Clearly, evaluation of LV and arterial function and their close interaction may provide a comprehensive assessment of the hemodynamic status of HD patients both in the interdialytic period and during volume depletion [12].

These findings underscore the complex interplay between fluid management, LV function, and arterial stiffness in dialysis patients. As dialysis treatments continue to evolve, a better understanding of these mechanisms will be essential for developing strategies to improve cardiovascular outcomes for kidney failure patients. Non-invasive tools, such as echocardiography and speckle-tracking techniques, are proving to be valuable in assessing these parameters, facilitating timely interventions aimed at minimizing the cardiovascular risks associated with chronic dialysis [5].

## Impact of hemodialysis on left ventricular morphology/function

### Cardiac hypertrophy in hemodialysis patients

LVH is the most common cardiac abnormality observed in patients with kidney failure and is an independent predictor of survival [1, 2]. Approximately 80% of HD patients exhibit increased LV mass, reflecting the high prevalence of cardiac remodeling in this population [3]. In patients with kidney failure, both pressure and volume overload are prevalent, contributing to an increase in LV mass [13]. Thus, the increase in LV mass in patients with kidney failure may result from wall thickening, LV chamber enlargement, or a combination of both. Chronic pressure overload in dialysis patients leads to an increase in the relative wall thickness resulting in concentric hypertrophy, while volume overload primarily causes LV dilation leading to eccentric hypertrophy [14]. However, accurate classification of LVH as either eccentric or concentric can be challenging in HD patients due to the cyclic fluctuations in extracellular fluid volume and electrolyte balance [15].

Hemodynamic factors, such as arteriovenous fistulas, secondary anemia, and intravascular volume expansion caused by salt and fluid retention are key contributors to volume overload. Moreover, other non-hemodynamic mechanisms may contribute to LV remodeling and hypertrophy. Hyperphosphatemia and vitamin D deficiency may play a role in regulating the growth and differentiation of cardiac myocytes [4]. Additionally, the partial regression of LVH with ACE-inhibitors highlights the involvement of the tissue and cardiac renin-angiotensin system in the development of LVH [16]. Elevated plasma endothelin levels have also been linked to LVH in kidney failure, supported by animal studies showing endothelin receptor antagonists reducing myocardial hypertrophy [17]. Hypoalbuminemia in kidney failure arises from multiple contributing factors, such as diminished liver production of albumin, protein losses during dialysis, inadequate nutritional intake, fluid overload, and other mechanisms related to the chronic kidney disease. Although hypoalbuminemia is often associated with reduced oncotic pressure and thus lower effective circulating volume, in the context of kidney failure it frequently coexists with volume overload and LV dilation [18–21]. However, the mechanism of the impact of hypoalbuminemia on the LV geometry is still unclear. Hyperparathyroidism, although implicated, remains controversial, as parathyroidectomy has shown minimal effects on cardiac structure and function [22]. Over time, pathological LVH may progress due to collagen deposition, fibrosis, and calcification [2].

Left ventricular hypertrophy is a well-established prognostic factor in patients with kidney failure. A study by Silberberg et al. demonstrated that LVH, as assessed by echocardiography, significantly influences survival outcomes in individuals initiating kidney replacement therapy. Patients in the highest quintile of LV mass index had a 3.7-fold increased risk of both all-cause and cardiac mortality compared to those in the lowest quintile, underscoring LVH as a critical independent predictor of survival in kidney failure [2]. Beyond baseline LVH, changes in LV mass index over time also play a crucial role in patient prognosis. A prospective study involving HD patients, which measured LV mass index twice over an average interval of 18 months, found that fluctuations in LV mass index were strongly associated with adjusted risks for both all-cause mortality and major adverse cardiovascular events [23]. Additionally, the structural geometry of LVH further impacts outcomes. A retrospective study of 175 chronic HD patients revealed that LV geometry and inferior vena cava diameter significantly influence cardiovascular risk and overall survival. Specifically, individuals with a large inferior vena cava diameter and eccentric LVH faced the highest risk of cardiac events, while those with a large inferior vena cava diameter and concentric LVH had the greatest risk of both cardiovascular and all-cause mortality. These findings emphasize the need for close monitoring and targeted interventions to manage LVH and volume status, and to improve survival in kidney failure patients [13].

Several studies suggest that intensive HD may contribute to a reduction in LV mass, potentially lowering the risk of adverse cardiac events. For instance, the Frequent Hemodialysis Network trial, a randomized clinical trial involving 245 patients, demonstrated that both short daily and nocturnal HD schedules resulted in LV mass reductions of 14 g (10%) and 11 g (8%), respectively, compared to the conventional thrice-weekly regimen [24]. Since reduction of LVH is linked to a lower risk of cardiovascular complications such as heart failure, post-myocardial infarction remodeling, and sudden arrhythmic death, further research is needed to explore the potential of intensive HD in minimizing cardiovascular risk [25].

### Impact of hemodialysis on left ventricular systolic function

Many studies report that cardiac function is affected by HD. A study by Wang and colleagues on 40 patients showed that the LV end-diastolic volume, LVEF and global longitudinal strain significantly decreased after HD. In this study, LVEF was assessed before and after HD sessions using the Simpson’s method with apical 4- and 2-chamber views, and global longitudinal strain was evaluated using 2D speckle-tracking echocardiography [5]. Another comparative study involving 30 patients with kidney failure and 30 healthy controls demonstrated that two-dimensional speckle-tracking echocardiography is more sensitive than conventional echocardiography in detecting subtle systolic and diastolic dysfunction in LVH patients, even when LVEF appears normal [26]. Moreover, global longitudinal strain is regarded as a more reliable predictor of cardiovascular mortality than echocardiographic LVEF in patients undergoing chronic HD [27].

Kidney failure patients who regularly undergo HD sessions experience acute preload changes, which consequently affect preload-dependent echocardiographic parameters. Deformation parameters have been claimed to be load-independent, however conflicting results have been reported on the changes of strain measurements, such as global longitudinal, circumferential and radial strain of the myocardium after HD. While some studies indicate a significant reduction of these parameters after HD, others showed similar values of global longitudinal strain before and after HD which suggests preload-independence [28–30].

Apart from the acute changes induced by HD on LV function, in the long-term HD drives progressive LV remodeling and dysfunction, typically associated with a gradual decline in LVEF. Even a modest drop of LVEF, more than 5% over several years of HD, is linked to higher mortality, while paradoxical small increases—often reflecting volume overload—also predict worse survival. Consequently, serial echocardiographic evaluation of LVEF is indispensable for timely identification and management of cardiovascular risk in this population [31].

Ventricular dysfunction is a known complication of transient myocardial ischemia, even after blood flow is restored. In patients with pre-existing LVH and interstitial fibrosis, HD-induced intravascular volume reduction triggers hemodynamic shifts, reflected in blood pressure and heart rate variations, and activates injury pathways, including high-energy phosphate depletion, microvascular hypoperfusion, impaired neural response, and inflammation [32]. These factors contribute to HD-induced ischemia, or myocardial stunning, leading to cardiac dysfunction. In 2009, Burton and colleagues found that 64% of 70 patients exhibited myocardial stunning during HD, which was defined as a reduction in wall motion greater than 20% from baseline in more than two regions [33]. Moreover, Assa et al. found that 27% of 105 patients undergoing HD developed regional LV systolic dysfunction, with 17 patients showing dysfunction as early as 60 min after dialysis initiation. The key factors associated with this dysfunction were male sex, a higher LV mass index, and pre-existing LV dysfunction. However, dialysis treatment-related factors, such as changes in blood volume, electrolytes, or acid–base parameters, were not linked to LV dysfunction. Furthermore, during follow-up, HD-induced regional LV dysfunction was identified as an independent risk factor for all-cause mortality, as the association remained significant even after adjusting for important prognostic factors, including age, sex, dialysis history, diabetes, cardiovascular risk factors, ultrafiltration volume, LV mass index, and predialysis LV systolic function [34]. These changes suggest that HD in patients with kidney failure may lead to LV dysfunction. This is also supported by the finding of impaired LV systolic function, as assessed by strain measurements, due to acute volume reduction after HD [35].

### Impact of hemodialysis on left ventricular diastolic function

Diastolic dysfunction has been shown to provide a significant additional prognostic value in predicting both all-cause mortality and cardiovascular-related death in HD patients [6]. While changes in diastolic function can partly be attributed to preload and afterload reduction, non-volume-related factors may also play a role [36]. Since coronary perfusion occurs during diastole, HD-related diastolic dysfunction may exacerbate myocardial ischemia, creating a cycle of myocardial stunning and further dysfunction [37]. Additionally, electrolyte imbalances—such as fluctuations in calcium, magnesium, and uncontrolled hyperphosphatemia—have been linked to diastolic impairment [38, 39]. A reduction in central aortic pressure during HD may further contribute to the development of diastolic dysfunction [40].

According to the American Society of Echocardiography and the European Association of Cardiovascular Imaging, four echocardiographic variables can be recorded to assess whether LV diastolic function is normal or abnormal. The four variables include mitral annulus *e*′ velocity (septal *e*′ < 7 cm/s, lateral *e*′ < 10 cm/s), average *E*/*e*′ ratio > 14, left atrial volume index > 34 mL/m^2^, and peak tricuspid regurgitation velocity > 2.8 m/s. From these parameters, left atrial pressure can be estimated [41].

In the study by Wang et al., LV diastolic function was evaluated using the aforementioned echocardiographic parameters before and after HD (within 24 h) (Table 1) [5]. This study found a significant reduction in both E-wave velocity and E/A ratio, whereas the decrease in A-wave velocity was not statistically significant. Furthermore, there was a significant decrease of *e*′ at the septal side of the mitral annulus (6.45 ± 1.88 vs. 5.77 ± 1.63 cm/s, *p* < 0.001), while the lateral annulus velocities showed no significant difference before and after HD (Table 1). The average value of the septal and lateral *e*′ also decreased significantly after HD (7.10 ± 2.14 vs. 6.53 ± 1.98 cm/s, *p* = 0.003). Additionally, the average *E*/*e*′ ratio decreased from 12.54 ± 4.08 before HD to 11.28 ± 4.52 after HD (*p* = 0.049), which was probably due to a greater reduction in E compared to *e*′ velocity. The decline in both E and *e*′ velocities suggests that mitral annular velocities are also preload-dependent. In addition, left atrial volume indexed by body surface area declined significantly after the HD session (30.22 ± 9.80 vs 35.55 ± 12.61 mL/m^2^, *p* < 0.001). The fourth variable, tricuspid regurgitation velocity, also showed a statistically significant decline after HD session (260.1 ± 36.5 vs. 242.3 ± 32.2 cm/s, *p* = 0.002) [5].

**Table 1:** Conventional echocardiography and pulsed Doppler measurements pre- and post-HD

Parameters	Pre-HD	Post-HD	*p* value
*E* (cm/s)	82.22 ± 20.13	72.43 ± 18.32	< 0.001***
*A* (cm/s)	94.91 ± 22.38	94.11 ± 19.96	0.672
*E*/*A*	0.90 ± 0.27	0.79 ± 0.23	< 0.001***
*e*′ septal (cm/s)	6.45 ± 1.88	5.77 ± 1.63	< 0.001*
*e*′ lateral (cm/s)	7.83 ± 2.59	7.33 ± 2.42	0.058
*e*′ average (cm/s)	7.10 ± 2.14	6.53 ± 1.98	0.003**
*E*/*e*′ average	12.54 ± 4.08	11.28 ± 4.52	0.049
LAVi (mL/m^2^)	35.55 ± 12.61	30.22 ± 9.80	< 0.001*
TRV (cm/s)	260.11 ± 36.54	242.37 ± 32.22	0.002**

*E*, peak early diastolic trans-mitral flow velocity; *A*, peak late diastolic trans-mitral flow velocity; *e*′ lateral, early diastolic velocity at the lateral mitral annulus; *e*′ septal, early diastolic velocity at the septal mitral annulus; LAVi, left atrial volume indexed to body surface area; TRV, Tricuspid regurgitation velocity [5]

***p < 0.001, **p < 0.01, vs. pre-HD

Recent studies have highlighted limitations in using mitral annulus velocities, particularly the E/e′ ratio, for assessing LV diastolic function [42]. This method estimates global LV diastolic function based on the assumption that one or multiple measurement sites reflect overall LV relaxation. However, in dialysis patients, significant LV diastolic dyssynchrony is frequently observed, reducing the accuracy of this approach [43]. Additionally, left atrial pressure can influence the early phase of LV filling, further complicating its reliability [44]. To address these limitations, LV global diastolic strain rate measurement using two-dimensional echocardiography speckle-tracking analysis has been demonstrated to be a more effective alternative, particularly in individuals with relatively preserved LVEF or regional wall motion abnormalities [45]. This measurement reflects the overall performance of all LV segments, remains independent of loading conditions, and accounts for initial LV size. For instance, a study involving 77 participants, of whom 59.7% were on HD, found a higher prevalence of diastolic dysfunction (defined by a ratio of diastolic early wave velocity to the global diastolic strain rate during isovolumic relaxation ≥ 236) compared to conventional diastolic dysfunction assessments (48% vs. 39%). These findings suggested that speckle-tracking-derived strain rate analysis might provide a more reliable evaluation of LV diastolic function in this patient population [42].

## Arterial changes in hemodialysis patients

Atherosclerosis and arterial thromboembolisms are among the most common causes of cardiovascular complications in individuals undergoing kidney replacement therapy [46, 47]. In addition to risk factors related to kidney failure (such as age, hypertension, smoking, diabetes, male gender, and insulin resistance), patients with renal dysfunction face unique contributors to atherosclerosis. These include symptoms attributable to kidney failure, dyslipidemia, calcium-phosphate imbalances, malnutrition, and increased cytokine activation [48].

Arterial remodeling in patients with chronic uremia is characterized by arterial stiffening, primarily driven by an elevated incremental elastic modulus and increased intima-media thickness [8, 9]. These changes are associated with reduced arterial distensibility, increased pulse wave velocity, and premature return of wave reflections [10, 11]. Arterial stiffness is independently linked to increased mortality in patients with chronic kidney disease [49]. It results from structural changes in vessel walls, particularly an imbalance between collagen and elastic fibers [50]. Arterial stiffness results in lower diastolic blood pressure, which affects coronary perfusion. On the other hand, the rigidity of arteries elevates central blood pressure, thereby increasing LV afterload. All together, these effects contribute to the development of hypertension, ischemic heart disease, and heart failure [51, 52]. Additionally, arterial stiffness contributes to fluctuations in blood pressure during dialysis, as documented by a correlation between endothelial dysfunction and intradialytic hypotension, as well as between arterial stiffness and intradialytic hypertension [53].

For many biomedical specialists, pulse wave velocity is the primary quantitative measure of arterial stiffness, representing the speed at which the arterial pulse travels through the arterial wall [54]. In clinical settings, pulse wave velocity is typically calculated using the formula PWV = ∆*L*/∆*t*, where ∆*L* denotes the distance between two measurement sites, and ∆t represents the time taken for the arterial pulse to travel from the proximal to the distal site [50]. Given that the aorta is the body's primary elastic artery, aortic pulse wave velocity—or even segmental aortic pulse wave velocity—is considered the most informative measure. The gold standard for pulse wave velocity measurement involves the use of an invasive pressure catheter; however, due to complexity, invasiveness, high cost, and ethical limitations the implementation of this method is restricted (Table 2). Among noninvasive alternatives, ultrasound or MRI-based pulse wave velocity measurements are available, but the high cost, logistic challenges, and limited temporal resolution prevent their widespread application in the general population.

**Table 2:** Comparison of techniques for the measurement of arterial stiffness (modified from Segers et al. [50])

Methods	Advantages	Disadvantages
Pressure catheters	Gold standard for measuring PWV in the aorta	Complexity, invasiveness, high cost, and ethical limitations
Magnetic resonance imaging, ultrasound of aorta	Noninvasive, accurately measures aortic pathology length	High cost, logistical challenges, and limited temporal resolution
Carotid-femoral PWV	Noninvasive, affordable, and suitable for large-scale population studies	Needs skilled personnel and offers less accurate measurement of PWV
Brachial-ankle, finger-toe PWV	Fast, affordable, and noninvasive	Significant uncertainty in correlation with aortic PWV
Single cuff	Fast, affordable, and noninvasive	Not possible to measure PWV based on unverified model assumptions
Ultrasound technologies for local PWV measurement	Noninvasive, enables direct evaluation of material stiffness, and allows measurements at various pressure levels	Costly ultrasound technology, dependent on ultrafast imaging, not fully integrated into commercial systems, still in the research phase with validation ongoing, and applicable only to superficial arteries

PWV, pulse wave velocity

The best available alternative option for aortic pulse wave velocity is carotid-femoral pulse wave velocity, which determines transit times based on signals recorded at the carotid and femoral arteries [50]. Although this method is cost-effective and can be applied to large populations, it requires trained personnel, and provides less precise path length and distance measurements (Table 2). Various devices have been developed to measure pulse wave velocity at more peripheral sites, such as brachial, ankle, or thigh, as well as photoplethysmography sensors on the fingers and toes [55, 56]. While these methods are noninvasive, quick, and inexpensive, they involve measurement sites far from the aorta. This introduces significant uncertainty regarding the exact path the pulse wave follows through elastic and muscular arteries, making it difficult to accurately correlate these values with aortic pulse wave velocity. Additionally, some devices claim to derive pulse wave velocity using only a single brachial cuff pressure recording [57]. While these devices are noninvasive, automated, fast, and cost-efficient, it is not physically feasible to determine pulse wave velocity from a single pressure waveform at one location. As a result, the values provided by such devices may be based on models with varying levels of validity [54].

Finally, high-resolution echo-tracking, usually at the site of the common carotid artery, can be used to evaluate local arterial stiffness in addition to pulse wave velocity (Table 2). More particularly, a study involving 58 patients on chronic HD revealed that carotid stiffness parameters, assessed using high-resolution echo-tracking, may predict all-cause mortality in HD patients [58]. Nevertheless, pulse wave velocity requires precise timing measurements between arterial sites (e.g., carotid to femoral artery), which can be affected by factors such as heart rate, arterial location, and the technique used for measurement [59]. This process can be more time-consuming and may be difficult to standardize across different practitioners and settings.

## Ventriculo-arterial coupling: pathophysiological concept and noninvasive assessment

### Calculation of ventriculo-arterial coupling

A comprehensive evaluation of cardiac function requires assessment not only of LV performance but also its coupling with the large arterial system, a relationship defined as ventriculo-arterial coupling [60]. Introduced by Sunagawa et al. in the early 1980s, ventriculo-arterial coupling is defined as the ratio of arterial elastance (Ea), a measure of the arterial system's effective afterload, to end-systolic elastance (Ees), a reflection of LV contractility [61]. This definition provided the framework for assessing the mechanical interplay between the heart and arterial system.

**Fig. 1:**
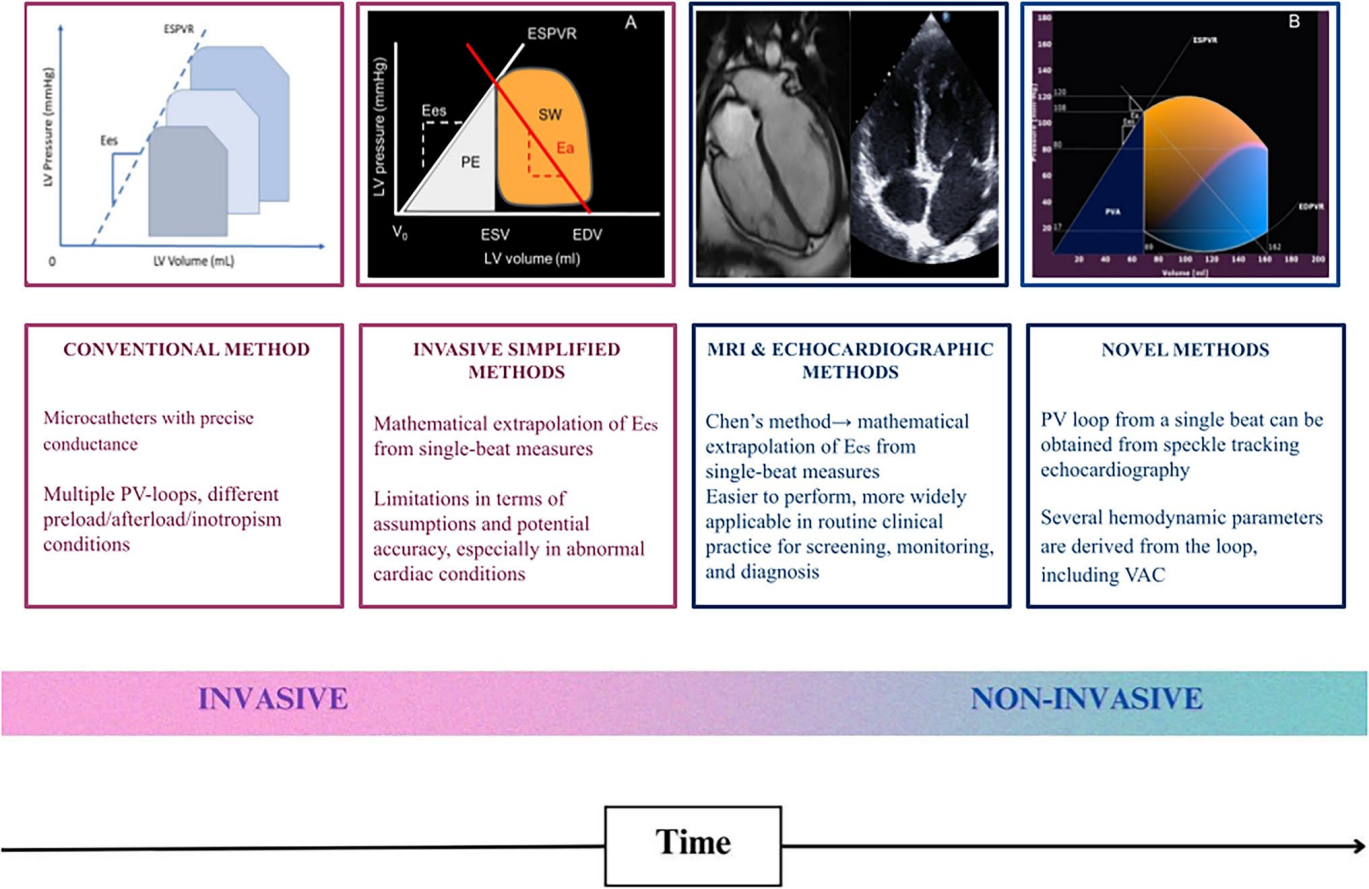
Chronological development of conventional, invasive simplified, and non-invasive methods for the evaluation of ventriculo-arterial coupling modified from Gamarra et al. [62]

The assessment of ventriculo-arterial coupling has evolved from invasive catheter-based techniques to advanced non-invasive imaging. Initially, high-fidelity conductance microcatheters and multiple pressure–volume loops were used for precise but complex ventriculo-arterial coupling evaluation. Later, simplified mathematical extrapolation methods allowed single-beat estimations. With the advent of cardiac magnetic resonance imaging and echocardiography, non-invasive approaches gained popularity. Today, speckle-tracking algorithms provide high-quality, semi-automated ventriculo-arterial coupling assessment (Fig. 1). The gold standard for measuring ventriculo-arterial coupling has traditionally involved direct recording of ventricular pressure and volume across various cardiac load conditions (multiple-beat) through cardiac catheterization. However, this approach is significantly limited by ethical concerns for human research and logistical challenges in its routine clinical application [63]. Researchers have since developed non-invasive approaches using echocardiography, magnetic resonance imaging, and arterial tonometry [63, 64]. These advancements have expanded the applicability of ventriculo-arterial coupling assessment, making it more feasible for routine clinical use. Through complex mathematical calculations, these methods allow end-systolic elastance to be extrapolated from the information obtained in a single cardiac cycle, avoiding the need for an invasive approach or for the characterization of multiple *P*/*V* loops. The shift toward non-invasive methods has also enabled dynamic ventriculo-arterial coupling evaluation, allowing clinicians to analyze how coupling responds to varying physiological states, such as exercise or pharmacological stress.

**Fig. 2:**
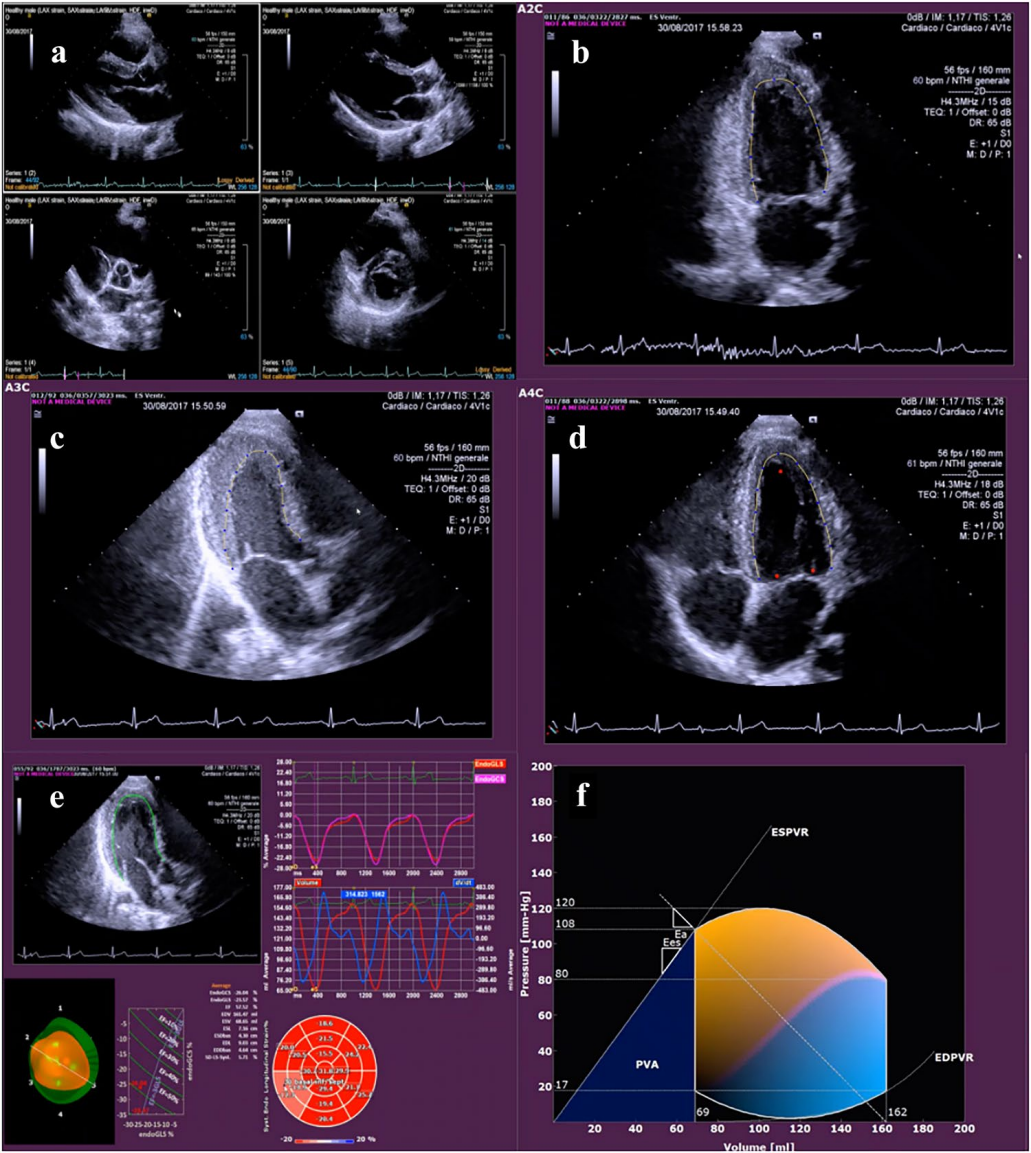
The steps from the echocardiographic images acquisition to the *P/V* loop reconstruction. The process includes: **a** acquisition of 2D echocardiographic images (apical 4-chamber, 2-chamber and long-axis); **b**–**d** analysis of the echocardiographic images by endocardial border delineation and speckle tracking (QStrain, Medis BV, Leiden, NL); **e** display of strain parameters; **f** reconstruction of the *P/V* loop using the single-beat algorithms [66]

Non-invasive single-beat approach to measuring arterial elastance and end-systolic elastance was introduced by Chen et al. [65], which involves blood pressure measurements from an arm cuff and echo-Doppler parameters. Currently, *P*/*V* loop from a single beat can be obtained from speckle-tracking echocardiography or feature-tracking cardiac magnetic resonance imaging using advanced software based on mathematical models [66]. The steps for generation of *P/V* loop from echocardiographic images are represented in Fig. 2. The *P/V* loop is reconstructed from the end-systolic pressure–volume relationship and the end-diastolic pressure–volume relationship). The end-systolic pressure–volume relationship is calculated using the equation: *P* = Ees (*V* − *V*_0_), where Ees represents the ventricular elastance calculated on the basis of the LV end-systolic pressure (*P*_es_) (estimated as 90% of the brachial systolic pressure) and the end-systolic volume (*V*_es_) [67]. The reference volume *V*_0_ = *V*_es_ − (*P*_es_/Ees) is then the intercept with the zero-pressure axis. This approach is widely applied in literature and it is considered appropriate for evaluating the ventriculo-arterial coupling [68]. The end-diastolic pressure–volume relationship is used to reconstruct the *P*/*V* loop, yielding a more precise depiction of ventricular function and is expressed as *P* = *αV*^*β*^, where the two parameters *α* and *β* are estimated as proposed by Klotz et al. on the basis of the end-diastolic volume (*V*_ed_) and end-diastolic pressure (*P*_ed_) [69], which is estimated noninvasively using the ratio *E*/*e*′ measured by Doppler or computed directly from the speckle tracking information using mass conservation and mitral valve size. Once the end-diastolic and end-systolic *P*/*V* relationships are identified, a *P*/*V* loop can be depicted for the entire cycle. The *P*/*V* loop is made by four branches in the pressure–volume plane:

1. Systolic contraction is represented as a curved trajectory from (*V*_ed_, *P*_es_) to (*V*_es_, *P*_es_), with a peak pressure at *P*_es_
2. Isovolumic relaxation is depicted as a vertical descent from (*V*_es_, *P*_es_) to (*V*_es_, *P*_ed_).
3. The diastolic filling curve starts from the (*V*_es_, *P*_ed_) and progresses toward the (*V*_ed_, *P*_ed_).
4. Isovolumic contraction is illustrated as a vertical rise from (*V*_ed_, *P*_ed_) to (*V*_ed_, *P*_es_).

The main properties of the *P*/*V* loop can be described by a series of parameters. In addition to end-systolic elastance, arterial elastance is evaluated as the ratio between end-systolic pressure and stroke volume, Ea = *P*_es_/(*V*_ed_ − *V*_es_), and from the ratio of the two elastance values (arterial elastance/end-systolic elastance) the ventriculo-arterial coupling can be calculated. The energetic properties are summarized in terms of stroke work (SW) given by the area of the *P*/*V* loop, the potential energy (PE) given by the area of the triangle made by the end-systolic pressure–volume relationship and the *P*/*V* loop, which is given by PE = 1/2 *P*_ed_ (*V*_es_ − *V*_0_), the total *P/V* area (PVA = SW + PE), and the work efficiency (WE = SW/PVA).

Recently, Toktarbay et al. have reviewed invasive and non-invasive methods for assessing ventriculo-arterial coupling [70]. Their findings highlighted the strengths and limitations of each approach. For instance, cardiac magnetic resonance was noted for its high precision, while echocardiography offered greater accessibility and widespread applicability. The study also emphasized the potential for future advancements, particularly in computational modeling and machine learning. These technologies could transform ventriculo-arterial coupling assessment by enabling patient-specific predictions and personalized therapeutic strategies, paving the way for more tailored and effective cardiovascular care.

Ventriculo-arterial coupling values carry significant clinical meaning. An elevated arterial elastance/end-systolic elastance ratio denotes ventricular–arterial uncoupling, which correlates with increased arterial stiffness, concentric LV hypertrophy, and pathological remodeling. Aging and hypertension exacerbate this uncoupling by raising arterial load and thickening the LV wall, whereas exercise interventions (e.g., cardiac rehabilitation or aerobic training) lower the arterial elastance/end-systolic elastance ratio—thereby restoring coupling, augmenting stroke work, and improving energetic efficiency [71]. Accordingly, ventriculo-arterial coupling is a powerful marker of cardiovascular health and offers prognostic insight across a range of clinical conditions.

Ventriculo-arterial coupling assessment extends beyond HD to conditions like heart failure with reduced ejection fraction, where it provides valuable prognostic information on risks of hospitalization and mortality [72]. It may also aid in determining the optimal timing for valve replacement in aortic stenosis patients [73]. In coronary artery disease-related ischemia, ventriculo-arterial coupling measurements can guide clinicians in gauging ischemic severity and selecting the most appropriate revascularization strategy. Regular ventriculo-arterial coupling monitoring can therefore assist in long-term management and prognostication in coronary artery disease [74]. However, its broader implementation is limited by technical complexity and variability in noninvasive measurement methods. As a result, clinicians often rely on more accessible echocardiographic indices—such as LV volumes, global longitudinal strain, pulse wave velocity—all of which, while useful, do not capture arterial function and carry their own limitations. The shift from invasive to noninvasive ventriculo-arterial coupling techniques has broadened its clinical accessibility, and ongoing innovations are anticipated to further optimize ventriculo-arterial coupling evaluation and solidify its role in everyday practice.

### Ventriculo-arterial coupling changes during hemodialysis

Data on changes of ventriculo-arterial coupling parameters after a HD session are scanty (Table 3). In 2013, researchers from Italy assessed ventriculo-arterial coupling in 15 patients with kidney failure after HD [75]. The study revealed that end-systolic elastance decreased, while arterial elastance remained unchanged. Consequently, ventriculo-arterial coupling significantly increased. According to the authors, these changes occurred due to an acute reduction in preload and blood pressure after the HD session, without any clinically significant influence on LV contractility and ventriculo-arterial coupling. Moreover, the study demonstrated that LV end-diastolic and end systolic volumes, and LV mass are also preload-dependent due to the previously mentioned mechanisms. According to Chen et al., an increase in blood pressure between HD sessions is related to the gain in body weight and fluid volume in patients with kidney failure [76]. Their hypothesis suggests that the end-systolic elastance parameter can predict how much blood pressure will rise depending on the weight gain. This is because, as the authors explain, in these patients there is an increase in the stiffness of both the left ventricle and the arteries, which amplifies the effect of fluid volume on blood pressure.

**Table 3:** Summary of the key findings on ventriculo-arterial coupling changes during HD

Study	Population	Key finding	Clinical implications
Sasso et al. (2013)	Patients with kidney failure on chronic HD (*n* = 15)	LVEDV, LVESV, and LV mass decreased after HD Ees decreased, while Ea remained unchanged Ventriculo-arterial coupling significantly increased, still remaining in the normal range	The changes in Ees and ventriculo-arterial coupling after HD are primarily due to the acute decrease in volume status (preload) and blood pressure HD has no clinically significant effect on intrinsic LV contractility or ventriculo-arterial coupling
Zuo et al. (2023)	Patients with kidney failure on chronic HD (*n* = 317)	Blood pressure, LVEDV, SV, LVEF, and systemic vascular resistance index decreased significantly after HD Both Ea and Ees increased Ventriculo-arterial coupling remained relatively stable	The rise in Ees primarily reflects passive stiffening The increase in Ea after HD may be partially due to greater arterial stiffness Ventriculo-arterial coupling appears to be largely load-independent
Obokata et al. (2017)	Patients with kidney failure on chronic HD (234)	Ees, PRSW, GLS, *S*′, Ea/Ees, *E*/*e*′ and LVEF were associated with outcome EDV and arterial afterload parameters did not show an association with the endpoint	LV contractility and ventriculo-arterial coupling were independently correlated with adverse outcomes in HD patients Ventriculo-arterial coupling added prognostic significance beyond clinical scoring and ejection fraction

E, peak early diastolic trans-mitral flow velocity; *e*′, peak early diastolic velocity at the mitral annulus; E_a_/E_es_, arterial elastance/end-systolic elastance; HD, hemodialysis; GLS, global longitudinal strain; LV, left ventricular; LVEDV, left ventricular end-diastolic volume; LVEF, left ventricular ejection fraction; LVESV, left ventricular end-systolic volume; PRSW, preload recruitable stroke work; S', peak systolic velocity at the mitral annulus; SV, stroke volume

In a recent study involving 317 patients on long-term maintenance HD, several significant alterations were observed in cardiovascular function following HD (Table 3) [77]. Compared to pre-HD measurements, there was a marked decrease in systolic blood pressure, mean blood pressure, pulse pressure, end-diastolic volume, and cardiac output. Specifically, mean arterial pressure decreased by 6.7%, end-systolic pressure dropped by 5.5%, and stroke volume decreased by 17.5%. However, no significant changes were observed in heart rate or end-systolic volume. Additionally, markers such as arterial elastance, systemic vascular resistance index, and end-systolic elastance all showed increases. Notably, ventriculo-arterial coupling remained stable, suggesting that the interaction between ventricular and vascular function was largely unaffected by HD. Furthermore, the study highlighted that ventriculo-arterial uncoupling (arterial elastance/end-systolic elastance > 1.0) was associated with a higher incidence of LVH and poorer cardiac function, as evidenced by lower *S*′, lower LVEF, and reduced end-systolic elastance. Patients with ventriculo-arterial uncoupling also tended to have a shorter duration of HD therapy. These findings suggest that ventriculo-arterial coupling may serve as a reliable, load-independent marker for assessing global cardiovascular function and stratifying cardiovascular risk in HD patients.

The only study on the value of ventriculo-arterial coupling as a prognostic indicator in patients undergoing HD showed that end-systolic elastance, arterial elastance/end-systolic elastance, global longitudinal strain, *S*′, *E*/*e*′ and LVEF were associated with poor outcome, while end-diastolic volume and arterial afterload parameters did not show an association with the endpoint [12]. Furthermore, nested Cox regression models revealed that arterial elastance/end-systolic elastance offered independent, incremental predictive value over models based on AROii score, LVEF or *E*/*e*′ ratio. Notably, the predictive utility of arterial elastance/end-systolic elastance remained significant even after adjusting for global longitudinal strain. This suggests that evaluating ventriculo-arterial coupling could improve risk prediction and stratification in dialysis patients.

**Fig. 3:**
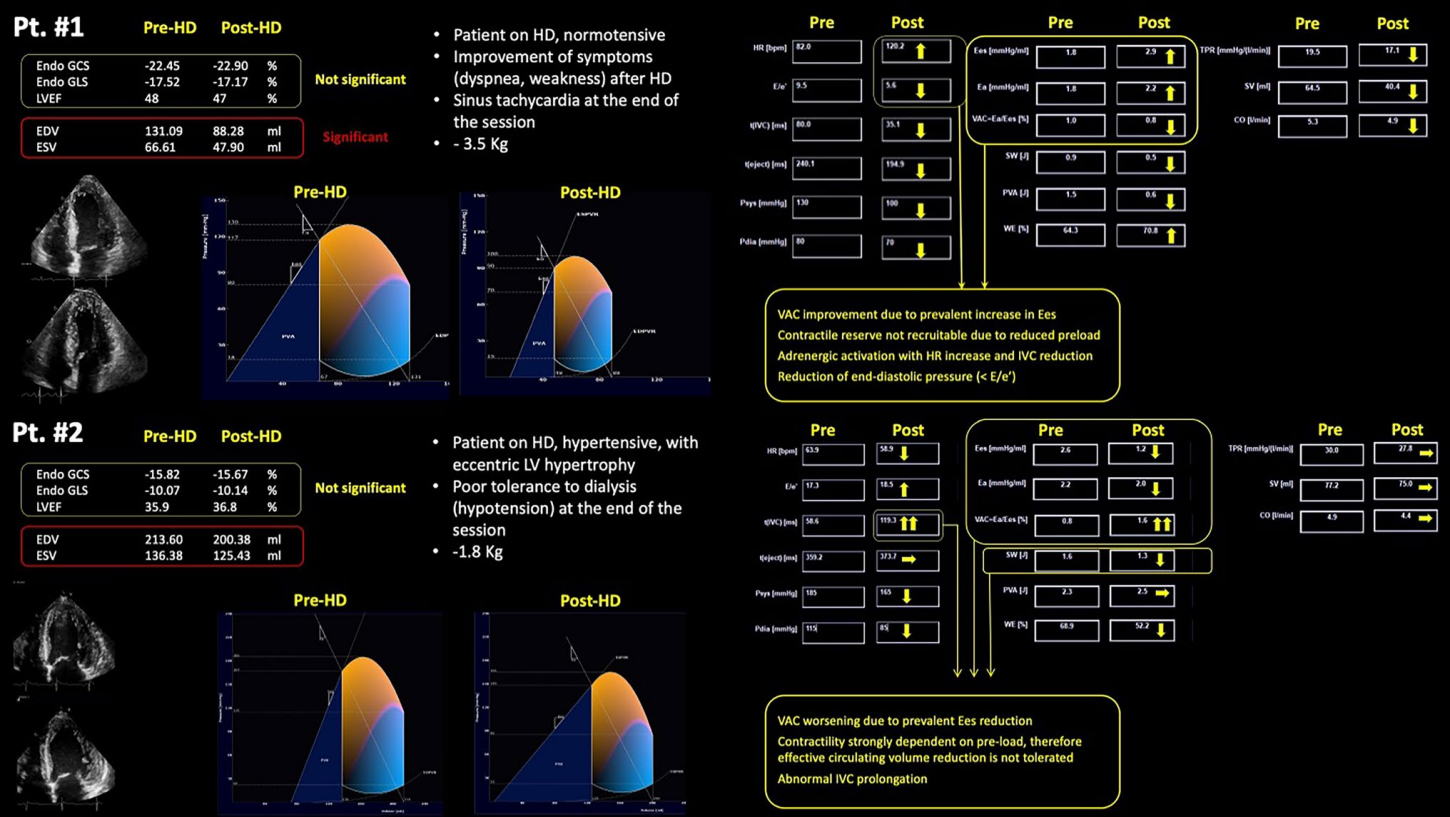
Echocardiographic images of two patient cases with kidney failure on chronic HD (from Salustri et al., [78])

Given the limited number of studies examining the prognostic value of ventriculo-arterial coupling in HD patients, we have designed a study protocol to investigate intradialytic changes and the prognostic significance of ventriculo-arterial coupling in patients with kidney failure [78]. This study employs a single-beat approach and advanced software based on a mathematical model to generate *P*/*V* loops from speckle-tracking echocardiography images. Figure 3 presents two case examples from this study. In Patient #1, 3.5 L of fluid were removed during the HD session, resulting in improved ventriculo-arterial coupling due to an increase in end-systolic elastance (from 1.8 to 2.9 mmHg/mL).

In contrast, Patient #2 experienced poor tolerance following the removal of 1.8 L of fluid, accompanied by symptoms of hemodynamic instability at the end of the HD session. Echocardiographic evaluation revealed a deterioration in ventriculo-arterial coupling due to a decrease in end-systolic elastance (from 2.6 to 1.2 mmHg/mL).

## Conclusions

Cardiovascular complications in HD patients are multifactorial, driven by structural and functional changes in the left ventricle, arterial stiffness, and the dynamic fluid shifts inherent to HD. LVH, influenced by both hemodynamic and metabolic factors, remains a key predictor of adverse outcomes, with its geometry playing a crucial role in cardiovascular risk stratification. Additionally, HD-induced fluctuations impair both systolic and diastolic function, contributing to increased morbidity and mortality. The interplay between LV performance and arterial structure, as captured by ventriculo-arterial coupling, further underscores the complexity of cardiovascular management in these patients. A deeper understanding of these mechanisms is essential for refining dialysis protocols and developing strategies to mitigate cardiovascular risks, ultimately improving long-term outcomes for patients with kidney failure.
